# Otx2-PNN Interaction to Regulate Cortical Plasticity

**DOI:** 10.1155/2016/7931693

**Published:** 2016-01-06

**Authors:** Clémence Bernard, Alain Prochiantz

**Affiliations:** Center for Interdisciplinary Research in Biology, CNRS UMR 7241/INSERM U1050, Labex Memolife, Collège de France, 11 Place Marcelin Berthelot, 75005 Paris, France

## Abstract

The ability of the environment to shape cortical function is at its highest during critical periods of postnatal development. In the visual cortex, critical period onset is triggered by the maturation of parvalbumin inhibitory interneurons, which gradually become surrounded by a specialized glycosaminoglycan-rich extracellular matrix: the perineuronal nets. Among the identified factors regulating cortical plasticity in the visual cortex, extracortical homeoprotein Otx2 is transferred specifically into parvalbumin interneurons and this transfer regulates both the onset and the closure of the critical period of plasticity for binocular vision. Here, we review the interaction between the complex sugars of the perineuronal nets and homeoprotein Otx2 and how this interaction regulates cortical plasticity during critical period and in adulthood.

## 1. Introduction

During postnatal development, anatomical and functional plasticity of neural circuits allows the cerebral cortex to adapt to the environment, as cortical connections can be remodeled by physiological activity. These windows of learning, or critical periods, are needed to establish an optimal neural representation of (and adaptation to) the surrounding environment. Several sensory, motor, linguistic, and psychological abilities can only be acquired during these periods [1], since plasticity is very limited outside them, in particular in the adult when circuits and synapses have been consolidated. Critical periods have been observed in various systems and across species [2], but since the pioneering work of Wiesel and Hubel 50 years ago in cats, the critical period has been primarily studied in the binocular visual cortex. It was shown at the time that the inputs from the two eyes compete when they first converge onto individual neurons in the binocular zone of the primary visual cortex [3]. This leads to a physiological and anatomical cortical representation of the relative inputs contributed by either eye [4]. During the critical period, monocular deprivation, the extended closure of one eye, produces a loss of cortical response to the deprived eye and a gain in the input of the open eye [5]. This sensitivity to monocular deprivation is restricted to the critical period that begins at postnatal day (P) 20 in rodents (about 1 week after eye opening), peaks at P30, and rapidly declines over the next days [6]. In humans, imbalanced inputs during this critical period result in a neurodevelopmental disorder called amblyopia. Indeed, improper timing of critical periods is responsible for many central nervous system pathologies, possibly including certain psychiatric diseases [7].

Many molecular factors have been implicated for the onset and the closure of the critical period. Binocular interactions are detected by the integrated action of local excitatory and inhibitory connections in the visual cortex. This excitatory/inhibitory balance is dynamically adjusted by cortical circuits where inhibitory connections develop later than the excitatory ones [8]. As an optimal excitatory/inhibitory balance is required for plasticity, critical period onset is triggered by the maturation of local inhibitory circuits [4, 9]. More specifically, critical period onset is triggered by the maturation of a subset of GABAergic inhibitory interneurons, the fast-spiking parvalbumin interneurons (PV-cells), located in layer IV of the cerebral cortex [10]. Precocious maturation of GABAergic innervation is prevented during the precritical period by factors such as *α*-2,8-polysialic acid bound to the neural cell adhesion molecule (PSA-NCAM) [11]. In response to sensory input, critical period onset is triggered when factors such as brain-derived neurotrophic factor (BDNF [12, 13]) or neuronal activity-regulated pentraxin (NARP [14, 15]) promote PV-cell maturation. This triggers a sequence of structural and molecular events that lead to circuit rewiring and physiological consolidation. During the critical period, layer IV PV-cells are gradually enwrapped by a specialized extracellular matrix giving rise to the perineuronal nets (PNNs) that surround the cell soma and proximal dendrites [16, 17]. PNNs are enriched in complex sugars called glycosaminoglycans (GAGs) and constitute a highly organized structure composed of hyaluronic acid, link proteins, proteoglycans, and tenascin-R [18, 19]. Physiologically, PNNs are part of the molecular brakes that progressively decrease plasticity and eventually close the critical period. Indeed, an emerging view is that the brain is intrinsically plastic and that adult plasticity is dampened by molecular brakes that limit excessive rewiring after critical period closure [20]. However, this is a reversible process and plasticity can be reopened after critical period closure, either by reinstalling lower levels of inhibition [21–23] or by lifting the molecular brakes (e.g., by disrupting the PNNs [24]). Opening windows of plasticity in the adult is of therapeutic interest [25], given that it has been used to cure amblyopia in rodents [26–31]. This review discusses how the PNN extracellular matrix interplays with homeoprotein Otx2 to regulate visual cortex plasticity and how interfering with this interaction can reopen windows of plasticity in the adult.

## 2. Otx2 Homeoprotein Transfer Regulates the Critical Period for Ocular Dominance Plasticity

The transfer of homeoprotein Otx2 in the visual cortex during postnatal development is necessary and sufficient for the onset and closure of the critical period for ocular dominance plasticity in mice [32]. Homeoproteins are well-known transcription factors that play major roles during embryonic development. For instance, several homeoproteins (including Otx2) are fundamental in controlling the specification, maintenance, and regionalization of the vertebrate brain [33]. Homeoprotein transcription factors share a highly conserved DNA-binding domain called homeodomain, but many homeoproteins also share activities that extend beyond their classical transcriptional role. Indeed, they are paracrine signaling factors that transfer between cells due to the presence within the homeodomain of sequences necessary for their unconventional intercellular transfer: a secretion sequence “Δ1” [34] and an internalization sequence “penetratin” [35].

Otx2 is no exception and has noncell autonomous activity in the supragranular layers of the binocular visual cortex [32, 36]. When transferred from extracortical sources into the visual cortex during postnatal development, Otx2 is internalized preferentially by PV-cells: in the visual cortex, a majority of neurons containing Otx2 are GABAergic inhibitory interneurons and over 70% of them are PV-positive [32]. The time course of Otx2 accumulation in PV-cells parallels that of PV-cell maturation: Otx2 protein is barely detected in the primary visual cortex prior to critical period onset, is increasingly concentrated by PV-cells during the critical period, and persists in adulthood. Interestingly,* Otx2* conditional knock-down heterozygous mice have a delayed onset of ocular dominance critical period, suggesting that a 50% reduction in Otx2 protein is sufficient to alter PV-cell maturation [32]. Otx2 therefore not only accumulates in PV-cells but also promotes their maturation and consequently regulates the onset of the critical period of plasticity for binocular vision.

## 3. Otx2 Binds Sulfated Glycosaminoglycans of the PNNs

The preferential capture of Otx2 protein by PV-cells suggests the existence of Otx2-binding sites at the PV-cell surface. As PV-cells are gradually enwrapped by PNNs during the critical period, a strong association between Otx2 and PNNs is observed in layer IV of the adult visual cortex [30]. PNN hydrolysis with the enzyme chondroitinase ABC (ChABC), which digests the GAG chains and reactivates plasticity in the adult cortex [24], decreases endogenous Otx2 concentration in PV-cells [30]. Another study showed that a decrease in PNN formation due to redox deregulation prevents the internalization of Otx2 by PV-cells [37]. It was thus concluded that complex sugars of the PNNs participate in the specific recognition of Otx2 before its internalization. A short motif within Otx2 sequence (RKQRRERTTFTRAQL), which partially overlaps with the first helix of the homeodomain, possesses consensus traits of a GAG-binding domain [38] and is a requisite for the specific recognition of Otx2 by PNN-surrounded PV-cells. Indeed, while a full-length exogenous Otx2 protein injected in the visual cortex shows a preference towards PNN-enwrapped cells, an Otx2-AA protein, in which the arginine-lysine (RK) doublet is replaced by two alanines (AA), shows less preference for PNNs and is internalized by a wider range of cells [30]. Accordingly, when a synthetic peptide corresponding to this GAG-binding motif (RK-peptide) is infused into the visual cortex of adult mice, it competes with endogenous Otx2 and blocks its transfer into PV-cells. The reduced capture of Otx2 by PV-cells results in a downregulation of PV expression and PNN assembly, as if the maturation status of PV-cells was reversed to a critical period state. This “rejuvenation” was confirmed by the reopening of ocular dominance plasticity following infusion of the RK-peptide in the adult cortex and the ensuing recovery of visual acuity in amblyopic mice [30].

The sulfation pattern of glycan chains is thought to encode specific information for the binding of growth factors and morphogens, such as Wnt, Hedgehog, BMP, and FGF [39]. The sulfation pattern of PNNs differs from that of GAGs of the diffuse matrix and the three main types of GAGs present in the PNNs are the chondroitin sulfates (CS), heparan sulfates, and hyaluronic acid [18, 19]. Isothermal titration calorimetry experiments with commercial subtypes of chondroitin sulfates showed that the RK-peptide binds strongly to disulfated chondroitin sulfates CS-D and CS-E, has a lower affinity for CS-C and heparin, and shows no measurable binding to CS-A [30]. However, the affinities of GAGs for the full-length 32 kD protein may be different from the 15-amino-acid peptide as specificity is expected to be altered by the increased size and the probable structural changes [40]. Otx2 full-length protein binds to a synthetic hexasaccharide analogue of CS-E [41] and six monosaccharide units seem to be the minimum GAG chain length for Otx2 binding. Infusion of this CS-E analogue in the visual cortex of adult mice blocks Otx2 transfer in PV-cells [41], supporting the idea that binding to specifically sulfated GAGs is required for proper transfer of Otx2 into cortical PV-cells. Interestingly, the CS-E subtype is also required for the binding of the semaphorin Sema3A to the PNNs in the visual cortex [42, 43]. In addition, modification of the sulfation pattern of PNNs (using transgenic mice with a low 4S/6S ratio) reduces Otx2 accumulation in the PV-cells of the visual cortex [44]. These mice show extended ocular dominance plasticity in the adult, confirming both the role of Otx2 in the regulation of critical period in the visual cortex and the importance of a specific sulfation pattern for Otx2 binding.

## 4. A positive Feedback Loop between Otx2 and PNNs

Not only are the complex sugars of the PNNs necessary for Otx2 preferential transfer into PV-cells, but Otx2 is in turn involved in PNN assembly, both during the critical period and in the adult. Indeed, early Otx2 infusion in the visual cortex, before the onset of the critical period for ocular dominance, accelerates PNN expression leading to an early closure of plasticity [32]. In addition, in dark-rearing conditions that delay PV-cell maturation [45, 46], direct infusion of Otx2 in the visual cortex leads to increased amount of PNNs around PV-cells [32]. The opposite effect is observed in* Otx2* conditional knock-out heterozygous mice in which Otx2 protein amounts in the visual cortex are strongly reduced: these mice, which have a delayed ocular dominance critical period, also show a delay in the maturation of the PNNs [32]. It can thus be concluded that Otx2 transfer triggers the maturation of PNNs during postnatal development. In the adult, one of the main sources of cortical Otx2 is the choroid plexus. This structure, present in brain ventricles and responsible for the synthesis of cerebrospinal fluid, is an established site of Otx2 expression throughout life [31, 47]. In the adult mouse, Otx2 is secreted by choroid plexus epithelial cells into the cerebrospinal fluid, and knocking-down* Otx2* specifically in the adult choroid plexus decreases Otx2 cortical content [31]. This decrease in Otx2 is accompanied by a decrease in PV expression and PNN assembly. As already mentioned, this is also the case when Otx2 transfer is blocked at the level of the target cells in the adult cortex, by using the RK-peptide or the synthetic CS-E analogue: both infusions lead to a reduction in the number of PNNs surrounding PV-cells [30, 41]. Otx2 transfer in the adult therefore seems to be required to maintain the PNNs in a mature state.

**Figure 1:**
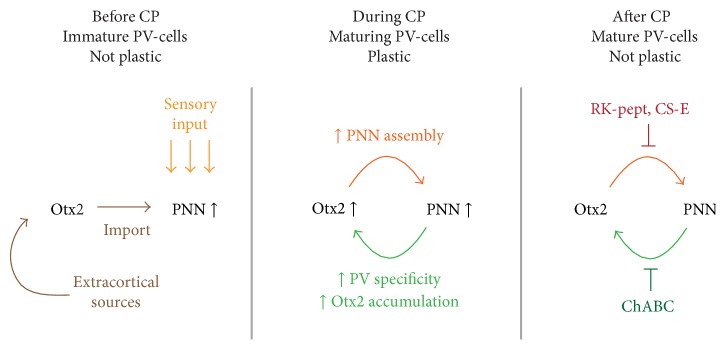
*Otx2-PNN feedback loop for critical period plasticity*. At critical period (CP) onset, sensory activity induces initial formation of the perineuronal nets (PNNs), allowing the internalization of extracortical Otx2 by PV-cells. During CP, the increasing PV-cell Otx2 content enhances PNN assembly. In turn, PNNs ensure the specific accumulation of Otx2 in the PV-cells. In the adult, the constant transfer of Otx2 into the PV-cells, due to the positive feedback loop between the homeoprotein and the PNNs, maintains a mature, consolidated, nonplastic state. Indeed, interfering with Otx2-PNN interaction in the adult (by injecting ChABC to remove PNNs or by infusing the GAG-binding domain of Otx2 (RK-peptide) or a CS-E analogue to block Otx2) reopens a window of plasticity in the visual cortex.

Both gain- and loss-of-function experiments indicate that Otx2 internalization enhances PNN assembly [30–32, 41]. The ongoing positive feedback of PNNs attracting Otx2, thus triggering their own continued maintenance throughout life, may serve to prevent plasticity in adulthood (Figure 1). Otx2 regulation of plasticity can therefore be explained by a two-threshold model: the critical period is triggered as Otx2 is first captured by PV-cells but then closes as maturing PNNs condense in response to Otx2 accumulation, thus permitting a constant accumulation of Otx2 by PV-cells [31]. However, the mechanisms through which Otx2 regulates the maturation and maintenance of the PNNs are yet unknown. Otx2 could modify the expression of members of the PNNs. For example, the homeoprotein has been shown to regulate the expression of extracellular matrix proteins such as tenascin-C and DSD-1-PG* in vitro* [48]. Otx2 could also regulate the expression of enzymes that modify the extracellular matrix such as the metallopeptidases Adamts, which are expressed by PV interneurons [49]. Otx2 molecular targets for PV-cell regulation are also unidentified. The fact that Otx2 transfer is necessary and sufficient to open plasticity at P20 and close it 20 days later and that blocking Otx2 is enough to reopen a window of plasticity in the adult cortex suggests a very general action of Otx2. Epigenetic changes have been linked to critical period and adult plasticity [29, 50, 51] and Otx2 could act at the epigenetic level to globally modulate PV-cell maturation. Beyond the understanding of plasticity mechanisms during postnatal development, identifying these plasticity targets of Otx2 could lead to the development of precise tools to reopen windows of plasticity in the adult.

## 5. Glycans Could Be Involved in the Recognition of Homeoproteins for Unconventional Transfer

GAG moieties vary considerably in size, in the number of disaccharides per core protein, and in the position and degree of modifications, primarily sulfation, allowing huge molecular diversity and structural complexity. Complex sugars are precisely distributed in the postnatal brain, suggesting the existence of a sugar code for specific protein distribution. Therefore, specific sugar epitopes may provide a sugar code for homeoprotein recognition. Sequences homologous to the GAG-binding domain identified in Otx2 are present upstream of the homeodomain of many homeoproteins [52]. GAG-binding sites are often not conserved between proteins of the same family. In the case of chemokines, this allows the specificity and selectivity of GAG-binding across members of the family [40, 53]. The fact that Engrailed, another homeoprotein, does not accumulate specifically in PV-cells when infused in the cortex [30] supports the idea of specific surface binding sites for homeoproteins and of a glycan code for homeoprotein transfer specificity. The identification of precise sugar sequences could lead to the development of novel substances, such as synthetic CS-E analogue [41], to specifically interfere with homeoprotein transfer.

Infusion of homeoproteins in the brain parenchyma requires a coinfusion of polysialic acid to allow their diffusion [30, 32, 54]. Otherwise, the homeoprotein cannot diffuse and is immediately taken up by cells close to the infusion site. In the case of Otx2, the presence of polysialic acid allows the diffusion of the protein until it meets the PNN-enwrapped neurons. This suggests that endogenous traveling Otx2 is associated with low-affinity glycans and, once in the cortex, transfers from the latter low-affinity glycans to high-affinity PNN-associated glycans.

## 6. Otx2-PNN Interaction Might Coordinate and Synchronize Cerebral Cortex Plasticity

PNNs surround PV-cells not only in the visual cortex but throughout the central nervous system and have been found in the barrel cortex, frontal cortex, amygdala, striatum, substantia nigra, hippocampus, cerebellum, and spinal cord [24, 55–62]. Interestingly, Otx2 is present in PV-cells across all cortical regions, demonstrating that this transcription factor has a noncell autonomous widespread distribution and gains access to PV-cells in most cortical areas that include sensory regions such as the auditory and somatosensory cortices [31]. This makes it tempting to speculate that this factor acts as a global regulator of PV-cell and PNN maturation for cerebral cortex plasticity during development and in the adult [31]. Otx2 transfer could therefore have a wide role in regulating sensory experience during postnatal development. In the visual system, Otx2 transfer is activity-dependent and this raises the question of how activity (e.g., in the visual pathway, the opening of the eyes) operates. The formation of PNNs is also activity-dependent [63] as sensory deprivation by dark-rearing (visual cortex [29]) or whisker trimming (barrel cortex [55]) decreases the number of PNN-bearing neurons. One hypothesis is that as activity-dependent critical periods open following the initial activity of the corresponding peripheral sensory organs, sensory activity regulates an initial PNN assembly allowing for the accumulation of Otx2 en route from the choroid plexus.

Otx2 has been found not only in PV-cells of sensory cortices, but also in structures governing more complex behaviors, such as the amygdala, cingulate, and limbic cortices [31]. PNNs have recently been involved in these regions for the regulation of several types of memory in adulthood. Digestion of PNNs has been shown to increase adult learning capacities in the auditory cortex and perirhinal cortex [64, 65]. Enzymatic removal of PNNs in the prelimbic cortex or in the amygdala of adult rats impaired acquisition and reconsolidation of drug-induced memories [66, 67]. In a mouse model for Alzheimer's disease, digestion of PNNs in the perirhinal cortex enhanced object recognition memory [68]. Considering Otx2 function in maintaining the mature structure of PNNs in the adult, blocking Otx2 transfer could be used to promote cognitive flexibility and enhance memory acquisition in neurodegenerative diseases, for instance. PNNs have also been involved in critical periods for these regions, for instance, for fear extinction in the amygdala. It is of particular interest that, in amygdala, PNNs assemble at the closure of critical periods for fear extinction [57]. Whereas young mice can permanently erase an acquired fear memory by extinction training, adult animals exhibit fear behaviors that are resistant to erasure. In the adult basolateral amygdala, PNN degradation by ChABC reopens a critical period during which fear memories are fully erased by extinction training [57]. Otx2 transfer could therefore also regulate complex functions related to the emotional and anxiety state of the animal. Several reports propose that some psychiatric diseases may find their origin, at least in part, in cortical dysfunctions that occur in a period that precedes the onset of puberty [7, 69–71]. Defective maturation of PV-cells has been reported in cortex of subjects with schizophrenia [72] and has been proposed as one of the causes of psychiatric phenotypes [73–77]. In support of this hypothesis, PNN density is reduced in the amygdala and in the entorhinal and prefrontal cortices of subjects with schizophrenia [78, 79]. Critical periods therefore not only are governing the postnatal development of sensory systems but also have been involved in more complex behaviors, including language [80]. The role of Otx2 in various cortical regions has yet to be confirmed but Otx2 noncell autonomous presence in these areas suggests that this signaling may contribute to the orchestration of cascading critical periods underlying sensory behaviors and higher cognition.

## 7. Conclusion

Otx2 homeoprotein accumulation in PV-cells driven by sensory experience triggers a critical period for plasticity. Otx2 transfer regulates the maturation of the PNNs around PV-cells, which eventually closes the critical period. PNNs, in turn, maintain a stable postcritical period state by attracting Otx2 throughout life resulting in a positive feedback loop. PNNs therefore not only are molecular brakes that limit morphological and physiological plasticity, but also can act as “receptors” controlling the concentration of molecular factors that regulate plasticity and modulate PV-cell function, such as Otx2 and Sema3A. A better understanding of the Otx2 and extracellular GAGs interplay requires the identification of the precise glycan sequence that binds to Otx2 in the PNNs and of the mechanisms through which Otx2 regulates PNN assembly, maturation, and/or maintenance. This could allow the development of new GAG-related therapeutic strategies to block Otx2 transfer and reopen windows of plasticity with the hope to cure neurodevelopmental diseases.
